# Calcium-Sensing Receptor Gene Polymorphisms (CASRV1 and CASRV2) and the Physical Activity Level of Men in Lower Silesia, Poland

**DOI:** 10.3389/fgene.2020.00325

**Published:** 2020-04-21

**Authors:** Aleksandra Zagrodna, Anna Ksia̧żek, Małgorzata Słowińska-Lisowska, Łukasz Łaczmański

**Affiliations:** ^1^Department of the Biological and Motor Basis of Sport, University School of Physical Education in Wrocław, Wrocław, Poland; ^2^Laboratory of Genomics & Bioinformatics, Institute of Immunology and Experimental Therapy, Polish Academy of Sciences, Wrocław, Poland

**Keywords:** bone, bone density, calcium-sensing receptor, gene polymorphism, physical activity, IPAQ, men

## Abstract

Calcium-sensing receptors (CASR) are a dimeric family of C-class G-protein-coupled receptors that play a crucial role in bone and mineral metabolism by regulating parathyroid hormone (PTH) secretion, skeletal development, and urinary Ca^2+^ excretion. Genetic factors mainly impact bone mineral density (BMD). However, many variable factors may affect bone health, including physical activity. The aim of our study was to investigate the potential associations between calcium-sensing receptor gene polymorphisms (CASRV1 and CASRV2) and the level of physical activity in adult males from Lower Silesia, a region in the south of Poland. A total of 428 adult male inhabitants of Lower Silesia were included in the study. Their physical activity was evaluated using the International Physical Activity Questionnaire. The CASRV1 (rs 1801725, G>T, A986S) and CASRV2 (rs 761486, T>G, non-coding region) polymorphisms were determined using polymerase chain reaction (PCR) and mini-sequencing. The polymorphisms were identified with GeneScan software ver. 3.1.2. We did not observe any statistically significant differences between the total energy expenditure (total MET) and the CASRV1 and CASRV2 polymorphisms. We did not find any association between the level of physical activity and the frequency of genotypes at the polymorphic locus of the calcium-sensing receptor genes CASRV1 and CASRV2. We found that the number of hours the subjects spent in a sitting position was unrelated to the genotypes at the polymorphic locus of the calcium-sensing receptor gene CASRV1. Based on our studies, we concluded that there were no associations between CASR and physical activity in the men inhabiting Lower Silesia in Poland. Our results do not suggest any influence of the assessed genetic factors in the population variability of the level of physical activity of adults.

## Introduction

Regular physical activity is one of the most effective means of maintaining good physical and mental health in humans. In recent years, multiple studies investigating the impact of physical activity on health have been funded by organizations such as the European Union, the World Health Organization, and the International Olympic Committee. Despite increasing expenditures on the promotion of active lifestyles, most populations fail to achieve the recommended level of daily physical activity (Laaksonen et al., 2001; Drygas et al., 2001, 2005; Varo et al., 2003; Adams and Schoenborn, 2006; Haskell et al., 2007; Lightfoot et al., 2018; Kurita et al., 2019). There is a growing need to also examine intrinsic factors that determine variations in individual levels of physical activity. For many years, researchers have been investigating the molecular determinants of spontaneous locomotor activity in animals (Lerman et al., 2002; Tou and Wade, 2002; Turner et al., 2005; Lightfoot et al., 2008). A number of publications has recently suggested that the level of physical activity in humans is significantly affected by genetic factors (Joosen et al., 2005; Stubbe et al., 2005, 2006; Lightfoot, 2011; Mitchell et al., 2016; Herbert et al., 2018). Studies on the human model conducted so far have revealed associations between the level of physical activity and the DNA sequence variability of the BDNF (brain-derived neurotrophic factor) gene (Bryan et al., 2007), the DRD2 (dopamine receptor D2) gene (Simonen et al., 2003), the LEPR (leptin receptor) gene (Stefan et al., 2002), the ACE (angiotensin-converting enzyme) gene (Winnicki et al., 2004), the MC4R (melanocortin-4 receptor) gene (Loos et al., 2005), and the CASR (calcium-sensing receptor) gene (Lorentzon et al., 2001).

The calcium-sensing receptor (CASR) on the surface of parathyroid cells is involved in the regulation of parathyroid hormone (PTH) secretion (Houillier et al., 2001) and is probably also involved in PTH gene expression and the proliferation of parathyroid cells (Misiorowski, 2003; Brown, 2007). In the kidneys, the CASR regulates Ca^2+^ and Mg^2+^ reabsorption and is involved in potassium and phosphate secretion as well as the mechanism of urine concentration (Ba and Friedman, 2004). In bone tissue, the CASR has been found in osteoblasts and osteoclasts, where it is involved in the regulation of bone remodeling – bone formation by osteoblasts and suppression of bone resorption in osteoclasts (Datta et al., 1989; Dvorak et al., 2007).

Six polymorphisms were found in the CASR gene – one in intron 5 just before exon 6 [IVS 5 – T88C] and the other five in exon 7 (coding region), with one in the sixth transmembrane (TM) domain [Ala826Thr], one in the seventh TM [C851S], and three in the C-terminal cytoplasmic tail (Ala986Ser [A986S], Gly990Arg [R990G], Gln1011Glu [Q1011E]) (D’Souza-Li, 2006). The CASR calcium receptor polymorphisms mainly concern a single nucleotide (SNP – Single Nucleotide Polymorphism). Three polymorphisms located on C-terminal plasma tail, namely A986S, R990G, and Q1011E, are best known and described. These polymorphisms demonstrate great clinical importance. In a study by Heath et al., 1996, polymorphisms A986S, R990G, and Q1011E were found in a group of 100 healthy Americans with incidences of 30%, 15%, and 10%, respectively. Cole et al. (1999) studied the polymorphism of the A986S calcium receptor. The study was carried out on 163 healthy women, and it was found that heterozygous subjects with AS polymorphism and homozygous SS subjects had higher serum calcium levels, while homozygous AA subjects showed lower Ca levels. The presence of A986S polymorphism was found in 16% of the subjects. In Cole et al. (2001) confirmed their findings on a group of 387 women from Canada. Studies on A986S, R990G, and Q1011E calcium receptor polymorphisms and serum levels of ionized calcium were also conducted on a group of 377 unrelated Caucasian subjects (184 men and 193 women). It was found that the incidence of polymorphisms was 24%, 4%, and 3%, respectively. The presence of even a single S allele in A986S polymorphism resulted in higher serum levels of ionized calcium. On the other hand, the presence of even a single G allele in the R990G polymorphism resulted in lower ionized calcium concentrations. In terms of Q1011E polymorphism, the QQ genotype group demonstrated higher ionized calcium levels compared to the QE genotype group (Scillitani et al., 2004). The effect of the Gly990Arg calcium receptor polymorphism (R990G) on parathormone secretion was also studied in chronically hemodialyzed patients. It was found that the presence of AA homozygote at the polymorphic site of the calcium receptor led to significantly higher parathormone secretion (Yano et al., 2000).

An association between CASR gene polymorphisms and level of physical activity has also been reported. A study conducted by Lorentzon et al. (2001) showed a significant association between physical activity and the A986S polymorphism of the calcium-sensing receptor gene. Girls with a serine-coding sequence at the polymorphic locus of the CASR showed, on average, one and a half hours less physical activity per week than those with alanine. Alfred et al. (2012) reported that the presence of the T allele at the polymorphic locus resulted in a lower grip force.

The obtained results may have an important cognitive aspect, as references show no data on possible relations between the polymorphism of the CASR calcium receptor gene and individual preferences for spontaneous physical activity in the male group. Research on the molecular basis of physical activity may also have a practical aspect. That is because there is a perspective of possible individual adaptation of physical activity promotion programs as an important element in preserving the health of specific groups of people. The aim of our study was to investigate the potential associations between CASRV1, CASRV2, and the level of physical activity in adult males from Lower Silesia, a region in the south of Poland.

## Materials and Methods

### Subjects

A total of 428 adult male inhabitants of Lower Silesia were included in the study. The mean age was 47.02 ± 12.49 years. Among the study population, 43.8% of the subjects had completed higher education, while 56.2% had a primary or secondary school education. Additionally, 83.5% of the subjects were professionally active and 16.5% were retirees. The anthropometric characteristics of the study population are presented in Table 1.

**TABLE 1:** The anthropometric characteristics of the study population.

	Mean value ± SD	Min–Max
Age (years)	47.02 ± 12.49	22–72
Body mass (kg)	84.34 ± 13.67	54–132
Height (cm)	175.67 ± 6.88	159–196
BMI (kg/m^2^)	27.33 ± 4.18	16–43
WHR	0.96 ± 0.07	0.74–1.20

*BMI, body mass index; WHR, waist–hip ratio.*

### Assessment of Physical Activity

The level of physical activity was evaluated using the International Physical Activity Questionnaire (IPAQ) Short Form (Craig et al., 2003). The intensity of activity was defined as energy expenditure and was expressed in METs (metabolic equivalents: 1 MET is equivalent to the consumption of 3.5 ml of oxygen per minute per kg of body weight) (Kent, 1994). Physical activity estimated with the IPAQ was finally expressed in MET-minutes/week, being a sum of individual energy expenditures during high-, intermediate-, and low-intensity activities (Ainsworth et al., 2000). The subjects were assigned to one of three levels of physical activity based on the IPAQ protocol. As the groups with low and intermediate levels of physical activity both adhered to a similar total energy expenditure, they were pooled into a single group for the purposes of the genetic testing and were labeled with the letter L (low).

### Data Collection

CASRV1 and CASRV2 levels were investigated in a group of 455 men. A blood sample from an arm vein was collected from each subject in a tube with EDTA as an anticoagulant and the samples were stored at −20°C. Genomic DNA was isolated using a column-based method (A&A Biotechnology). The CASRV1 (rs 1801725, G>T, A986S) and CASRV2 (rs 761486, T>G, non-coding region) polymorphisms were determined using polymerase chain reaction (PCR) and mini-sequencing (SNaPshot Kit, Applied Biosystems, United States). A PCR Core Kit (Qiagen) and the following sequence of primers (composition of the reaction mixture: 10x concentrated buffer, 1.5 mM of MgCl2, 200 nM of reaction primers, and 250 μM of dNTPs) were used for the CASRV1 polymorphism: primer 1 (Fw) 5′ AGCAAGAGCAACAGCGAAGA 3′, primer 2 (Rv) 5′GCGGGAGTAATGGCTGGTGT 3′; and for the CASRV2 polymorphism: primer 1 (Fw) 5′ ATCTGGGTTCTCTCTTCTGT 3′, primer 2 (Rv) 5′CAAGGAACTACAAAAGACCC 3′. A gene amplification reaction was carried out under the following conditions: initial denaturation at 95°C for 3 min, followed by 35 cycles at 95°C for 30 s, annealing at 55°C for 45 s, elongation at 72°C for 30 s, and final elongation at 72°C for 5 min. The amplification products were cleaned up by removing free nucleotides and oligonucleotide fragments with the enzymes Exo and SAP (Fermentas, United States). This step was followed by employing the mini-sequencing technique to detect the polymorphic loci. The mini-sequencing technique involves carrying out a polymerase reaction in the presence of a primer, a probe, and fluorescent dideoxynucleotides. For CASRV1, the probe was 5′GAGCTTTGATGAGCCTCAGAAGAAC 3′, while for CASRV2 the probe was 5′GTAGTGTGATGCCTCTGGCTTTGTTCTTTGCTCT 3′. Product separation and detection were then carried out. An ABI 310 Analyzer was used (Applied Biosystems, United States). The polymorphisms were identified with GeneScan Software ver. 3.1.2 (Applied Biosystems, United States).

### Ethics Statement

All subjects gave written informed consent in accordance with the Declaration of Helsinki for human subjects and the European Communities Council directive of November 24, 1986 (86/608/EEC). The protocol was approved by the Bioethics Committee of the University School of Physical Education in Wrocław, Wrocław, Poland, resolution number 18/2013.

### Statistical Analyses

Statistical analyses were performed using STATISTICA (StatSoft, Inc.), version 8.0.

All analyses were performed to correct for body mass index (BMI), waist-hip ratio (WHR), and smoking (yes/no).

Variables were first analyzed for normal distribution by the Shapiro–Wilk test.

The relationship between the polymorphism of the calcium receptor gene CASRV1 and CASRV2 and the level of physical activity expressed as low or high was analyzed using bipartite tables and the χ2 dependence test.

The Kruskal–Wallis test was used to assess the relationship between genotypes at the polymorphic site of the CASRV1 and CASRV2 calcium receptor gene, and the total energy expenditure, as well as the number of hours spent by the subjects in a sitting position.

The relationship between the physical activity level, the age of the subjects and the number of hours spent in a sitting position was assessed by mean of non-parametric tests using the Mann–Whitney *U* test. The relationship between the total energy expenditure and the age of the subjects and time spent in a sitting position was analyzed by estimating Spearman’s rank correlation coefficients.

A *p*-value < 0.05 was considered to be significant in all analyses.

## Results

Two polymorphisms were evaluated in the study: CASRV1 (rs 1801725, G>T, A986S) and CASRV2 (rs 761486, T>G, non-coding region).

The genotypes identified at the polymorphic locus were as follows: for CASRV1, G/G in 64.3% of the subjects, G/T in 32.6% of the subjects, and T/T in 3.1% of the subjects; for CASRV2, G/G in 6.1% of the subjects, T/G in 38.8% of the subjects, and T/T in 55.2% of the subjects.

The values of the mean total energy expenditure for the individual CASRV1 genotype variants were as follows: 5793.7 ± 7774.7 MET-min/week for G/G, 6487.6 ± 8505.6 MET-min/week for G/T, and 4141.7 ± 4469.5 MET-min/week for T/T (Table 2). The values of the mean total energy expenditure for the CASRV2 genotype variants were as follows: 7109.2 ± 8723.5 MET-min/week for G/G, 6145.1 ± 7818.1 MET-min/week for G/T, and 5708.2 ± 7909.9 MET-min/week for T/T (Table 2).

**TABLE 2:** Total energy expenditure (MET-total) and the *CASRV1* and *CASRV2* polymorphisms.

	TOTAL ENERGY EXPENDITURE (MET–min/week)					*p*-value
	Mean value ± SD	Lower quartile	Median	Upper quartile	Min–Max	
**CASRV1 genotypes**					
G/G	5793.7 ± 7774.7	1386.0	3225.0	6666.0	0–56790	0.75
G/T	6487.6 ± 8505.6	1191.0	2772.0	8346.0	0–45648	
T/T	4141.7 ± 4469.5	1386.0	2313.0	4452.0	198–12690	
G/T + T/T	6272.9 ± 8237.2	1230.0	2772.0	8064.0	0–45648	0.77
**CASRV2 genotypes**					
G/G	7109.2 ± 8723.5	1386.0	3598.0	7092.0	132–31140	0.68
T/G	6145.1 ± 7818.1	1480.0	3273.0	6024.5	0–42660	
T/T	5708.2 ± 7909.9	1306.5	2853.0	6826.5	0–56790	
G/G + T/G	6274.3 ± 7979.4	1428.0	3370.0	7092.0	0–42660	0.43

*p-value was determined by comparing medians.*

No statistically significant differences in total energy expenditure (total METs) between CASRV1 and CASRV2 were observed, even when we conducted the analysis in pooled groups: G/T + T/T at the polymorphic locus of the calcium-sensing receptor gene CASRV1 and T/G + G/G at the polymorphic locus of the calcium-sensing receptor gene CASRV2 (Table 3).

**TABLE 3:** Level of physical activity and frequency of genotypes at the polymorphic locus of calcium-sensing receptor genes CASRV1 and CASRV2.

Genotypes	CASRV1	
	LOW L (n)	HIGH H (n)
G/G	132	143
G/T	70	69
T/T	8	6
Total	210	218
χ^2^	0.63	
*p*-value	0.73	

	**CASRV2**		

G/G	12	14
T/G	79	89
T/T	118	116
Total	209	219
χ^2^	0.60	
*p*-value	0.74	

No statistically significant association between the level of physical activity and the frequency of genotypes at the polymorphic locus of the CASRV1 and CASRV2 were observed, even when we evaluated the presence or absence of the T allele at the polymorphic locus of CASRV1 and the presence or absence of the G allele at the polymorphic locus of CASRV2 (Table 3).

The number of hours the subjects spent in a sitting position was unrelated to genotype at the polymorphic locus of CASRV1 and was as follows: 6.3 ± 3.1 h/day for G/G, 6.2 ± 2.7 h/day for G/T, and 6.0 ± 3.9 h/day for T/T. No statistically significant correlation was observed between genotype at the polymorphic locus of CASRV2 and the number of hours spent by the subjects in a sitting position. The average number of hours spent in a sitting position in the groups with the T/T and the T/G genotypes at the polymorphic locus of CASRV2 were similar: 6.2 ± 3.1 h/day and 6.1 ± 2.9 h/day, respectively. The average number of hours spent in a sitting position was higher in the case of the G/G genotype at the polymorphic locus of CASRV2 was 7.3 ± 3.2 h/day. No statistically significant correlation was observed between the genotypes at the polymorphic locus of CASRV2 and the number of hours spent by the subjects in a sitting position.

## Discussion

In our study, we evaluated two polymorphisms of the CASR: CASRV1 (rs 1801725, G>T, A986S) and CASRV2 (rs 761486, T>G, non-coding region). We analyzed both the coding and non-coding regions of the gene. This may have significant exploratory implications, as the available literature includes reports suggesting that the genetic regulation is a result of combined effects of multiple transcription factors interacting with coding DNA regions or with sequences in the gene’s vicinity. Luque et al. (2004) showed that regulatory elements of gene expression may also be found in non-coding regions.

In the present study, we investigated the calcium-sensing receptor gene polymorphism CASRV1; the frequency of the G allele was 80.5%, while that of the T allele was 19.5%. As regards the calcium-sensing receptor gene polymorphism CASRV2, the frequency of the G allele was 25.7%, while that of the T allele was 74.3%. The distribution of frequencies of specific genotypes was as follows: in the case of CASRV1, it was 64.3% for G/G, 32.6% for G/T, and 3.1% for T/T; in the case of CASRV2, it was 6.1% for G/G, 38.8% for T/G, and 55.2% for T/T. In a study by Lorentzon et al. (2001) conducted on girls living in Sweden, the following distribution of the frequencies of specific genotypes of calcium-sensing receptor polymorphism A986S (designated as CASRV1 in our study) was observed: 71.1% for G/G, 25.8% for G/T, and 3.1% for T/T. This distribution was similar to the one we obtained in men from Lower Silesia. Similar distributions of specific genotypes at the polymorphic locus of CASRV1 were also reported by Cole et al. (1999) and Bollerslev et al. (2004). The frequencies of specific genotypes at the polymorphic locus of CASVR2 in the study subjects were as follows: 55% for T/T, 39% for T/G, and 6% for G/G. The fact that there are few reports on this polymorphism is noteworthy. The distribution of specific genotypes at the polymorphic locus of CASVR2 in a study published by Lips et al. (2007) and conducted on an English population aged 59 to 71 years was as follows: 58% for T/T, 35.7% for T/G, and 6.3% for G/G; this was also similar to that observed in the Polish population.

Our study did not show any statistically significant association between either CASRV1 or CASRV2 and the level of physical activity, whether expressed in METs or in more general categories (low vs. high). Lorentzon et al. (2001) assessed the level of physical activity in the study subjects using a Standardized Questionnaire and expressed it as the number of hours of physical activity per week. Those who did not have the S protein variant at the polymorphic locus of calcium-sensing receptor A986S showed a significantly higher level of physical activity (4.3 ± 2.6 h/day) than those who did have that allele (2.9 ± 2.6 h/day). These findings suggest that individuals with the G/G genotype at the polymorphic locus of calcium-sensing receptor A986S demonstrate a higher level of physical activity than those with the G/T or T/T genotypes. It should be noted, however, that the study by Lorentzon et al. (2001) was conducted on girls aged 16.9 ± 1.2 years on average, and as such, cannot be directly compared with a study conducted on a population of men aged 47.13 ± 12.46 years on average.

The study of Swedish girls also used a different method than ours for the evaluation of physical activity (Lorentzon et al., 2001). Bollerslev et al. (2004) conducted a study in which they analyzed 1,252 elderly women aged between 70 and 85 years. Physical activity was evaluated by hand grip force and the Timed Up and Go (TUG) test. The study found that individuals with the S protein variant at the polymorphic locus of calcium-sensing receptor A986S had a grip force that was nearly identical with those who did not have that allele (20.6 ± 4.4 kg vs. 20.5 ± 4.7 kg). Likewise, the results of the TUG test did not suggest any differences between the subjects with the S protein variant and those without it (10.0 ± 2.9 s vs. 9.9 ± 3.1 s). These findings fail to confirm any statistically significant association between the calcium-sensing receptor gene polymorphism at the polymorphic locus of A986S and the level of physical activity as determined by hand grip strength and the TUG test. A multicenter study was conducted on 11,239 English men between the ages of 52 and 90 years whose level of physical activity was determined using such performance tests as the hand grip strength test, the TUG test, the chair test, and the balance test. The subjects were considered “physically active” if they reported participating in sports at least once a month. Based on the test results, it was determined that individuals with the T allele at the polymorphic locus of calcium-sensing receptor gene A986S had significantly lower hand grip strength. As regards the other performance tests, no differences were observed between subjects with the T allele and those without it. Also, no association was shown between the CASR gene polymorphism and the level of physical fitness (Alfred et al., 2012).

Like other authors (Bryan et al., 2007; Karoly et al., 2012; García et al., 2018), we are aware that no significant differences should be expected to be present between variants of a single gene and such a complex pattern of behavior as one’s level of physical activity. In this context, it is justified to make attempts to design interdisciplinary models that would combine specific behaviors with the multiple factors these behaviors are affected by.

When interpreting the results, one should take into account the contribution of limiting factors that might have affected them. The majority of subjects comprising the study population were healthy men with a normal BMI (27.33 ± 4.18 kg/m^2^). It is worth noting that a considerable percentage of the study population consisted of men with a university degree, who typically are more aware of the positive impact of physical activity on health. Despite this fact, we didn’t notice a correlation with this knowledge and the real physical activity level of the study population. Our sample was derived from a genetically homogenous population. It should also be noted that the subjects completed the IPAQ on their own, and many studies have confirmed that when the level of physical activity is assessed by a questionnaire without a qualified interviewer, the respondents tend to overestimate their level of physical activity (Rzewnicki et al., 2003; Biernat et al., 2008, 2012). At the same time, it should be emphasized that the IPAQ is considered to be a very good tool for the assessment of physical activity among a population (Craig et al., 2003; Ekelund et al., 2006; Faulkner et al., 2006; Mäder et al., 2006; Maddison et al., 2007; Biernat et al., 2008; Ishikawa-Takata et al., 2008; Rangul et al., 2008; Hansen et al., 2014).

Based on the results of our study, we concluded that there is no correlation between CASR and physical activity in the men inhabiting Lower Silesia, Poland. The results do not confirm that this genetic factor has an influence on the population variability of the level of physical activity of adults. However, it should be noted that we studied only white men and generalizing our conclusions to other populations should be cautious. Future studies could explore this issue further by joining an analysis of CASRV1 and CASRV2 polymorphism with an evaluation of calcium and PTH levels.

## Data Availability Statement

The raw data supporting the conclusions of this article will be made available by the authors, without undue reservation, to any qualified researcher.

## Ethics Statement

The protocol was approved by the Bioethics Committee of the University School of Physical Education in Wrocław, Wrocław, Poland, resolution number 18/2013. The patients/participants provided their written informed consent to participate in this study.

## Author Contributions

AZ coordinated the research, carried out the data collection, and drafted the manuscript. AK helped draft the manuscript. MS-L participated in the experimental design and coordination and reviewed the manuscript. ŁŁ revised the article critically for important intellectual content. All authors have read and approved the final version of the manuscript.

## Conflict of Interest

The authors declare that the research was conducted in the absence of any commercial or financial relationships that could be construed as a potential conflict of interest.
